# *KRAS* Mutations in Circulating Tumor DNA for Lung Cancer Diagnosis: A Comprehensive Meta-Analysis

**DOI:** 10.3390/cancers18020250

**Published:** 2026-01-14

**Authors:** Karolina Buszka, Łukasz Gąsiorowski, Claudia Dompe, Anna Szulta, Michał Nowicki, Agata Kolecka-Bednarczyk, Joanna Budna-Tukan

**Affiliations:** 1 Department of Histology and Embryology, Poznan University of Medical Sciences, 60-781 Poznan, Poland; mnowicki@ump.edu.pl; 2 Doctoral School, Poznan University of Medical Sciences, 60-812 Poznan, Poland; claudia.dompe@student.ump.edu.pl (C.D.); anna.szulta@student.ump.edu.pl (A.S.); 3 Department of Educational Analytics and Continuing Professional Development, Faculty of Health Sciences, Poznan University of Medical Sciences, 60-812 Poznan, Poland; lgasior@ump.edu.pl; 4 Department of Immunology, Poznan University of Medical Sciences, 60-806 Poznan, Poland; akolb@ump.edu.pl (A.K.-B.); jbudna@ump.edu.pl (J.B.-T.); 5 Department of Anatomy and Histology, Collegium Medicum, University of Zielona Gora, 65-046 Zielona Gora, Poland

**Keywords:** *KRAS*, ctDNA, liquid biopsy, lung cancer, biomarkers, ddPCR, NGS, NSCLC

## Abstract

**Background**: Mutations in the *KRAS* gene play a pivotal role in lung cancer development and progression and are becoming increasingly important in therapeutic decision-making. The detection of these mutations in circulating tumor DNA (ctDNA) has attracted attention as a minimally invasive diagnostic approach. However, the accuracy reported in different studies varies widely. **Methods**: We conducted a systematic review and meta-analysis in accordance with the PRISMA-DTA guidelines. Eligible studies evaluated the detection of *KRAS* mutations in ctDNA in plasma or serum for lung cancer diagnosis and reported sufficient data to construct 2 × 2 contingency tables. Primary pooled estimates of sensitivity, specificity and likelihood ratios were calculated using aggregated 2 × 2 contingency tables. Additionally, a bivariate random-effects model was applied in a secondary analysis to investigate between-study heterogeneity. **Results**: Nine diagnostic study arms comprising 691 patients met the inclusion criteria. Across all datasets, there were 255 true positives, 19 false positives, 136 false negatives, and 281 true negatives. The pooled sensitivity was 65.2%, while the pooled specificity was 93.7%. The positive likelihood ratio was 10.35, and the negative likelihood ratio was 0.37, resulting in a diagnostic odds ratio of 28.0, which indicates strong rule-in capability. Sensitivity showed moderate heterogeneity across studies. In contrast, specificity demonstrated minimal heterogeneity. **Conclusions**: ctDNA-based detection of *KRAS* mutations demonstrates high specificity but moderate sensitivity for diagnosing lung cancer. These findings suggest that a *KRAS* liquid biopsy could be a valuable complementary diagnostic tool, particularly when a tissue biopsy is not possible or is inadequate, and it could support more personalized decision-making as analytical technologies continue to advance.

## Simple Summary

*KRAS* mutations are among the most common genetic alterations in lung cancer and can now be detected in circulating tumor DNA (ctDNA) via a straightforward blood test. These liquid biopsy approaches are appealing because they are minimally invasive and can provide genetic information when a tissue biopsy is not possible. However, the diagnostic accuracy of ctDNA in identifying *KRAS* mutations remains unclear. In this study, we conducted a systematic review of the available evidence and performed a meta-analysis of nine clinical studies involving 691 patients. Our findings suggested that the detection of *KRAS* mutations in ctDNA demonstrates very high specificity, meaning a positive result strongly indicates the presence of lung cancer. However, sensitivity was moderate and varied across studies. Overall, our findings suggest that *KRAS* ctDNA testing could support the decision-making process in diagnosis, but should complement, rather than replace, standard diagnostic procedures.

## 1. Introduction

Lung cancer remains one of the leading causes of cancer-related mortality worldwide. The prognosis is strongly dependent on an early and accurate diagnosis [1]. Although imaging techniques such as computed tomography and positron emission tomography can significantly improve disease detection, histopathological confirmation remains essential. However, obtaining adequate tissue samples can be difficult in patients with small peripheral lesions, inaccessible tumor locations, or severe comorbidities that make invasive procedures impossible [2]. These limitations have increased interest in minimally invasive diagnostic approaches that can provide molecular information without the need for tissue biopsies.

Liquid biopsy has become a promising complementary tool for detecting circulating tumor components, including circulating tumor DNA (ctDNA), circulating tumor cells (CTCs), extracellular vesicles, and tumor-educated platelets, in peripheral blood. ctDNA has emerged as a clinically relevant biomarker reflecting tumor-specific genomic alterations and correlating with tumor burden, stage, and treatment response [3,4]. Due to its short half-life, it is possible to monitor tumor dynamics in real time, making it suitable for assessing minimal residual disease, early relapse, and therapeutic resistance [5].

As many of these genomic alterations directly inform therapeutic decisions, particularly regarding the selection of targeted agents, accurately detecting them in ctDNA has become increasingly important at the interface of diagnosis and treatment. Targeted therapies, particularly tyrosine kinase inhibitors (TKIs), have transformed the treatment of advanced non-small-cell lung cancer (NSCLC) by providing improved response rates and tolerability for patients with actionable driver mutations, including alterations in the *ALK*, *ROS1*, *BRAF*, *MET*, and *KRAS* genes [6,7]. *KRAS* mutations are among the most clinically relevant of these biomarkers, and their detection in ctDNA offers a promising, minimally invasive approach to tumor genotyping. This information can be used to inform the use of emerging *KRAS* inhibitors [8,9,10]. The biological importance of *KRAS* mutations justifies their evaluation as diagnostic markers in liquid biopsies. However, published studies report highly variable sensitivity and specificity for blood-based *KRAS* detection, reflecting differences in tumor burden, ctDNA shedding dynamics, and assay methodology [11,12]. To address these inconsistencies, we conducted a systematic review and meta-analysis to evaluate the diagnostic accuracy of circulating *KRAS* testing in NSCLC. Our findings aim to clarify its potential diagnostic utility and support the evidence-based integration of ctDNA analysis into clinical practice.

## 2. Materials and Methods

This meta-analysis was conducted in accordance with the Preferred Reporting Items for Systematic Reviews and Meta-Analyses (PRISMA) guidelines, ensuring methodological rigor and transparency in the assessment of diagnostic test accuracy. The literature review was conducted using the PubMed/Medline, Scopus, Embase, and Cochrane databases to search for entries in English from 2005 to the date of the search (17 November 2025).

The following keywords were used in the search strategy, in the given order: “liquid biopsy”, “non-small cell lung cancer” and “*KRAS*”, which were combined using Boolean operators and supplemented with relevant Medical Subject Headings (MeSH) and free-text terms. Studies were included if they assessed *KRAS* mutations in the bloodstream using plasma or serum samples, and if they used histopathology or an accepted clinical diagnostic standard as a reference point. Studies also had to provide sufficient data to create a 2 × 2 contingency table. Both prospective and retrospective studies were included. Studies that evaluated ctDNA only for prognostic or therapeutic monitoring purposes, lacked extractable diagnostic data, or duplicated data from other publications were excluded.

Two reviewers independently assessed the eligibility of full texts after reviewing titles and abstracts, resolving disputes through discussion. The risk of bias and applicability concerns of the included studies were independently assessed by two reviewers using the QUADAS-2 tool. The following four domains were evaluated: patient selection, index test, reference standard and flow and timing. Each domain was judged as having a low, high or unclear risk of bias. Any disagreements were resolved by consensus. For each study included in the review, we extracted data on publication details, mutation targets, testing platform, sample size and diagnostic outcomes. These were classified as true positives (TPs; ctDNA correctly identifies mutations confirmed by the reference standard), false positives (FPs; ctDNA indicates mutations that are not confirmed by the reference standard), false negatives (FNs; ctDNA fails to detect mutations that are confirmed by the reference standard) and true negatives (TNs; ctDNA correctly indicates the absence of mutations that are confirmed by the reference standard). The final dataset comprised 9 study arms involving a total of 1535 patients, of whom 255 were true positives and 281 were true negatives. There were also 136 false negatives and 19 false positives. Sensitivity analyses were performed on studies that were assessed as having a high risk of bias in any QUADAS-2 domain. Many studies showed an unclear risk of systematic error due to an insufficient description of blinding procedures or positivity thresholds.

Diagnostic accuracy measures, including sensitivity, specificity, predictive values, accuracy and diagnostic odds ratio, were calculated at the study level. Combined measures were then calculated based on the aggregated data. This aggregated approach was used to generate transparent and reproducible primary pooled estimates that directly reflect the observed diagnostic outcomes. Additionally, a bivariate random-effects model (Reitsma model) was employed for secondary analysis, exploring between-study heterogeneity and the correlation between sensitivity and specificity. The between-study heterogeneity was evaluated using Cochran’s Q test and quantified using the I^2^ statistic and τ^2^ variance estimates. This approach enabled robust estimation of overall diagnostic performance while ensuring transparency and reproducibility of the methodology. Finally, forest plots were generated to show the sensitivity and specificity. A summary receiver operating characteristic (SROC) curve was then constructed using a bivariate random-effects model to illustrate the heterogeneity between studies and the diagnostic performance. Publication bias was assessed using Deeks’ funnel plot asymmetry test, as this is recommended for diagnostic test accuracy meta-analyses.

## 3. Results

### 3.1. Results of the Literature Search

**Figure 1:**
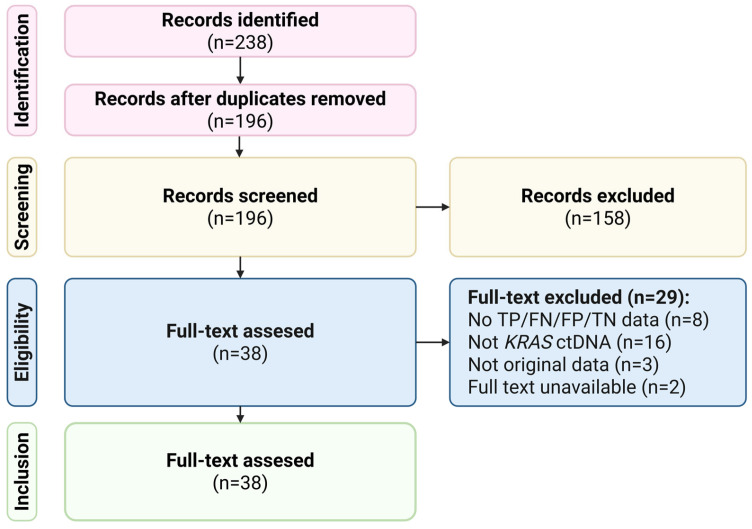
PRISMA flow diagram summarizing the study selection process. Created with www.BioRender.com (www.biorender.com, accessed on 16 December 2025).

A systematic search of the PubMed/MEDLINE, Scopus, Embase and Cochrane databases yielded a wide variety of publications evaluating the diagnostic value of circulating biomarkers (primarily circulating free DNA) in the detection of *KRAS* mutations in non-small-cell lung cancer. After removing duplicate records and conducting a screening of titles and abstracts, a subset of studies was reviewed in full. Following application of the predefined eligibility criteria, 9 study arms representing 691 patients were included in the quantitative synthesis. The PRISMA flow diagram illustrates the selection process from identification to inclusion (Figure 1).

### 3.2. Study Characteristics

The study arms included in the quantitative synthesis showed significant differences in terms of mutation targets, analytical platforms and sample sizes. All nine evaluated *KRAS* mutations. The analytical platforms used were quantitative PCR (qPCR), digital PCR, droplet digital PCR (ddPCR) and next-generation sequencing (NGS). These platforms are characterized by different analytical sensitivities and limits of detection. Sample sizes varied considerably, ranging from 22 to 189 individuals, reflecting a combination of small exploratory studies and larger, more extensive diagnostic cohorts. The aggregated dataset from all included study arms consisted of 255 true positives, 19 false positives, 136 false negatives, and 281 true negatives, resulting in a total evaluable population of 691 participants. This distribution provided a balanced foundation for estimating pooled diagnostic accuracy and assessing between-study variability (Table 1).

### 3.3. Quantitative Evaluation

Considerable variability in diagnostic performance was observed across individual study arms. Sensitivity ranged from 26.9% to 83.3%, reflecting variations in tumor burden, biological ctDNA shedding, assay sensitivity and positivity thresholds. By contrast, specificity was consistently high, ranging from 71% to 100%. Several datasets reported no false positives. This pattern is consistent with the high specificity of *KRAS* hotspot mutation detection technologies. Diagnostic odds ratios (DORs) also exhibited substantial variation, with study-level values ranging from approximately 10 to over 70. This highlights the variability in discriminatory capacity across different platforms and study populations. This heterogeneity emphasizes the importance of pooled meta-analytic estimates, as single-study accuracy metrics alone do not adequately capture true diagnostic performance in various settings. Table 2 summarizes the key diagnostic accuracy outcomes across the included studies.

### 3.4. Quantitative Evaluation (Meta-Analysis)

**Figure 2:**
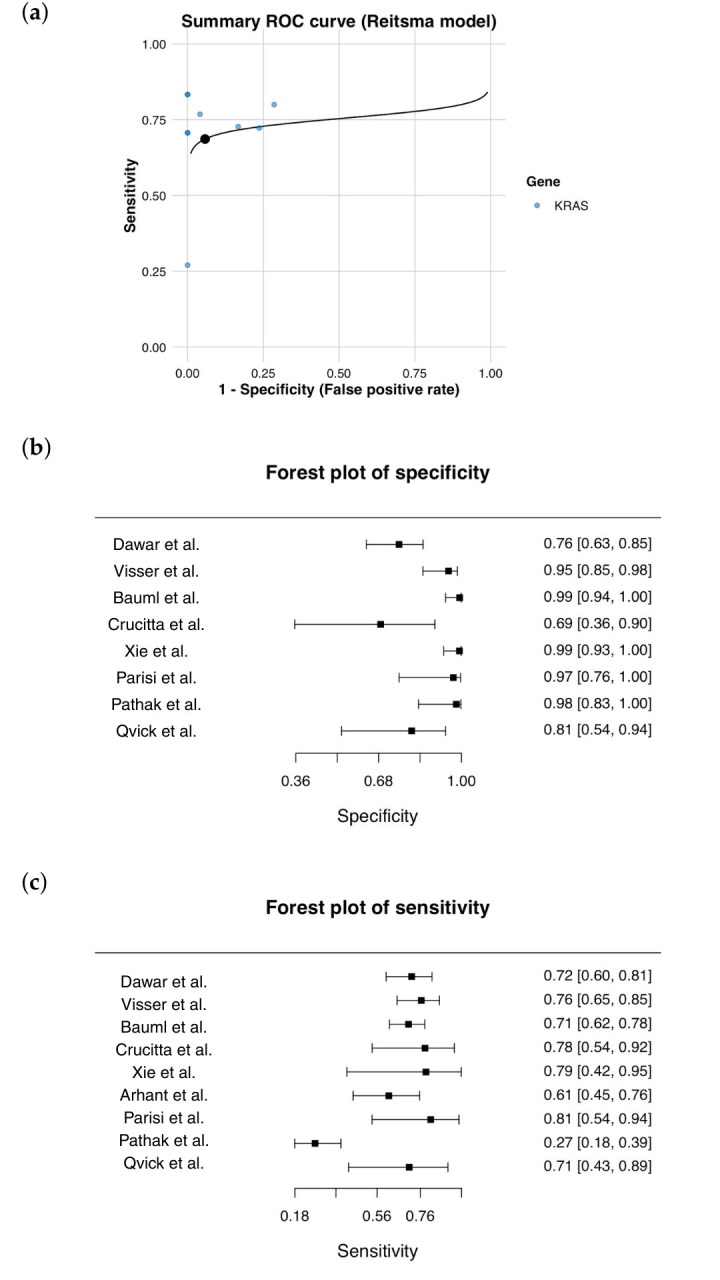
The summary receiver operating characteristic (SROC) curve and forest plots of diagnostic performance for *KRAS* mutation detection in ctDNA [13,14,15,16,17,18,19,20,21]. (**a**) SROC curve was generated using the Reitsma bivariate model. This shows the individual study estimates plotted against the pooled summary curve. The distribution of data points shows that specificity is consistently high, but sensitivity varies widely across studies. (**b**) A forest plot of study-specific specificity values with 95% confidence intervals illustrates that most studies reported near-perfect specificity. (**c**) A forest plot showing the sensitivity values and their 95% confidence intervals. This plot highlights substantial heterogeneity in the detection performance of the included datasets.

The diagnostic accuracy was synthesized by aggregating all the available true-positive, false-positive, false-negative and true-negative counts from the nine unique study arms. The combined dataset consisted of 255 true positives, 19 false positives, 136 false negatives and 281 true negatives. The pooled sensitivity of circulating *KRAS* mutation detection was found to be 65.2%, while the pooled specificity reached 93.7%. These results demonstrate an excellent ability to correctly identify individuals without lung cancer. The positive predictive value (PPV) was high at 93.1%, whereas the negative predictive value (NPV) was 67.4%. Likelihood ratio analysis further supported the strong ‘rule-in’ utility of the test, with an LR+ of 10.35 indicating that a positive result increases the probability of lung cancer by over tenfold. The LR- of 0.37 shows that while a negative test result reduces the likelihood of lung cancer, it does not eliminate it, which is consistent with the moderate sensitivity. The diagnostic odds ratio was 28.0, which is consistent with assays that offer strong diagnostic separation between cases and controls. ROC-style visualization revealed a close grouping of the study points at high specificity values, with a wide dispersion along the sensitivity axis (Figure 2). This pattern is typical of assays with high analytical specificity but variable sensitivity, resulting from biological and methodological differences across studies.

Substantial between-study heterogeneity was observed for sensitivity (I^2^ = 59.0%, τ^2^ = 0.49, Q = 19.49, *p* = 0.013), indicating considerable variability in detection rates across studies. In contrast, there was low heterogeneity for specificity (I^2^ = 4.5%, τ^2^ = 0.03, Q = 8.16, *p* = 0.418), which is consistent with the stable specificity generally observed across analytical platforms. The I^2^ value of 59.0% indicates moderate heterogeneity in sensitivity estimates across studies, whereas the I^2^ value of 4.5% suggests minimal heterogeneity for specificity. Consistently, the between-study variance (τ^2^) was higher for sensitivity (τ^2^ = 0.49), reflecting substantial variability between studies, while the τ^2^ value for specificity was low (τ^2^ = 0.03), indicating limited between-study variability. Cochran’s Q test confirmed statistically significant heterogeneity for sensitivity (*p* = 0.013), but not for specificity (*p* = 0.418). The secondary bivariate random-effects analysis using the Reitsma model revealed pooled sensitivities of 67.8% (95% CI 54.8–78.5%) and specificities of 92.5% (95% CI 81.1–97.2%). The summary receiver operating characteristic (SROC) curve showed that studies were clustered at high specificity values, but there was wide dispersion along the sensitivity axis. This reflects heterogeneous ctDNA detectability across study settings. The area under the SROC curve was 0.862, indicating good overall diagnostic accuracy. Assessment of publication bias using Deeks’ funnel plot asymmetry test did not provide evidence of significant small-study effects. Sensitivity analysis using a leave-one-out approach showed that the exclusion of any single study did not materially alter the pooled estimates, suggesting that the overall findings were robust to the influence of individual datasets. An exploratory subgroup analysis by analytical platform suggested that the pooled sensitivity was higher for next-generation sequencing-based assays than for PCR-based methods. However, specificity remained consistently high across platforms. Nevertheless, these findings should be interpreted cautiously due to the limited number of NGS-based studies.

### 3.5. Risk of Bias Assessment

The results of the methodological quality assessment, as determined by the QUADAS-2 tool, are summarized in Supplementary Figure S1. Most studies were judged to be at low risk of bias in the index test and reference standard domains. However, several studies showed an unclear risk of bias in the patient selection and flow and timing domains, primarily due to inadequate reporting of patient enrolment procedures, blinding or predefined positivity thresholds. Excluding studies with a high or unclear risk of bias in any QUADAS-2 domain from sensitivity analyses did not materially change the pooled diagnostic accuracy estimates, indicating the robustness of the results.

## 4. Discussion

This meta-analysis included nine study arms derived from nine unique studies and a total of 691 patients. It assessed the diagnostic performance of ctDNA-based detection of *KRAS* mutations in lung cancer. The aggregated contingency table across all datasets consisted of 255 true positives, 19 false positives, 136 false negatives, and 281 true negatives. These values correspond to pooled sensitivities and specificities of 65.2% and 93.7%, respectively, indicating that ctDNA assays achieve generally high specificity but only moderate sensitivity. This diagnostic pattern reflects the biological constraints associated with ctDNA shedding and technical variability across different detection platforms. By basing our discussion exclusively on original research, we provide an evidence-based interpretation of ctDNA performance in real clinical settings and the factors contributing to heterogeneity across studies. Although several studies were rated as having an unclear risk of bias in selected QUADAS-2 domains, this was mainly due to incomplete reporting rather than obvious methodological flaws, and it did not significantly impact the pooled estimates.

Biological determinants play a central role in determining the detectability of ctDNA. Early seminal work by Newman et al. demonstrated a strong correlation between ctDNA abundance and tumor volume in NSCLC, supporting the concept that ctDNA levels reflect total tumor burden [22]. Similarly, Bettegowda et al. found that ctDNA was detectable in most patients with advanced cancers, but at markedly lower rates in early-stage disease due to insufficient shedding [23]. Guibert et al. confirmed these findings in lung cancer specifically, reporting a pronounced drop in detection among early-stage tumors compared with advanced disease [24]. These results provide a biological explanation for the moderate pooled sensitivity observed in our analysis, even though specificity approached 100% in several study arms.

Unlike other oncogenic drivers, *KRAS* mutations are consistently more difficult to detect in ctDNA, primarily due to lower allele fractions and heterogeneous tumor shedding. Sacher et al. reported significantly lower concordance rates for ctDNA-based *KRAS* detection than for other genomic markers in a large prospective cohort. Newman et al. demonstrated that ddPCR reliably identifies *KRAS* mutations only when allele fractions exceed the lower limit of detection, emphasizing biological barriers to consistent ctDNA positivity [25]. These findings are particularly relevant in the context of *KRAS* G12C-targeted therapies, where accurate baseline genotyping is essential for identifying suitable patients. Clinical trials by Hong et al. and Skoulidis et al. reported promising therapeutic responses to sotorasib, yet also emphasized the importance of robust mutation detection for achieving the best possible clinical outcomes [26,27].

The moderate sensitivity observed in our meta-analysis is due to a combination of biological and technological factors. Newman et al. demonstrated that CAPP-seq can detect allele fractions below 0.1%, surpassing the capabilities of earlier technologies [22]. Razavi et al. demonstrated that NGS-based plasma genotyping detects mutations at higher rates than single-gene assays [28], and Chabon et al. reported that integrating multiple genomic features substantially improves sensitivity in the detection of early cancers [29]. Vessies et al. validated the BEAMing platform across multiple laboratories, revealing high analytical sensitivity, as well as notable inter-laboratory variability [30]. Taken together, these studies emphasize that assay performance is strongly influenced by analytical depth, platform characteristics, and laboratory standardization—factors that also contribute to the heterogeneity in *KRAS* detection observed across the studies included in our analysis.

Tumor heterogeneity is another key factor contributing to variation in ctDNA sensitivity. Landmark studies by de Bruin et al. and the TRACERx consortium [31] revealed significant spatial and temporal heterogeneity in NSCLC evolution [31,32]. As ctDNA is derived from all tumor sites, it can capture clonal diversity more comprehensively than a single-site tissue biopsy can. Studies by Murtaza et al. and Abbosh et al. have demonstrated that ctDNA can detect emerging mutations months before radiographic progression, enabling relapse to be revealed earlier than through standard clinical follow-up [33,34]. Gale et al. and García-Murillas et al. built on this concept by showing that ctDNA dynamics can predict recurrence more accurately and at an earlier stage than conventional imaging can detect minimal residual disease [35,36]. While these studies focus on monitoring rather than diagnostic accuracy, they highlight the biological complexity shaping variability in ctDNA sensitivity across *KRAS* studies.

Treatment response and disease monitoring also correlate strongly with ctDNA kinetics. Ricciuti et al. demonstrated that the dynamics of circulating tumor DNA can predict therapeutic responses and survival outcomes in patients with advanced NSCLC [37]. Chen et al. found that the detection of ctDNA during the perioperative period predicts recurrence in resected NSCLC [38]. While these findings highlight the prognostic and longitudinal value of ctDNA, they extend beyond the diagnostic scope of our meta-analysis.

Preanalytical variables can also have a significant impact on the performance of ctDNA assays. For example, Sherwood et al. demonstrated that delayed sample processing can lead to leukocyte lysis, which dilutes tumor-derived DNA and reduces sensitivity [39]. Parpart-Li et al. showed that stabilization tubes preserve ctDNA integrity and minimize background noise [40]. Hu et al. reported that clonal hematopoiesis introduces false-positive variants, highlighting the importance of distinguishing tumor-derived mutations from hematopoietic ones [41]. Such preanalytical challenges have likely contributed to the heterogeneity in sensitivity estimates observed in our pooled results. Although pooled specificity was generally high, some false-positive results were observed. Non-tumor-derived variants, including clonal hematopoiesis of indeterminate potential (CHIP), and pre-analytical factors may contribute to *KRAS* circulating tumor DNA ctDNA positivity. Therefore, ctDNA results should be interpreted alongside clinical findings and imaging and confirmed using an orthogonal method when feasible.

Recent methodological developments have continued to improve ctDNA detection. For example, Abbosh et al. demonstrated that incorporating phylogenetic tumor signatures into liquid biopsy workflows can enhance the detection of early-stage non-small-cell lung cancer [31]. Newman et al. introduced an analytical framework that combines fragment-omics with mutation profiling, substantially increasing detection rates [22]. These advancements suggest that limitations in current clinical practice, particularly the moderate sensitivity observed in *KRAS* studies, may diminish as sequencing technologies and error-suppression tools evolve.

Overall, the original research consistently supports several conclusions that align with our meta-analysis. Firstly, ctDNA has been shown to have a very high level of specificity for *KRAS* mutation detection, as reflected by the near-perfect specificity values observed in many of the included studies. Secondly, sensitivity is moderate and highly variable, influenced by tumor biology, assay design, and preanalytical factors—patterns that are directly reflected in our forest plots, funnel plot, and sROC curve. Thirdly, while our analysis focused on diagnostic accuracy at baseline, evidence suggests that ctDNA is also an effective tool for monitoring treatment response, identifying emerging resistance mechanisms, and capturing tumor evolution. As sequencing depth increases, error suppression improves, and integrative analytic frameworks advance, ctDNA is likely to become an increasingly important part of precision oncology for lung cancer.

## 5. Conclusions

This meta-analysis shows that, while ctDNA-based detection of *KRAS* mutations offers high diagnostic specificity, it has only moderate and heterogeneous sensitivity for identifying lung cancer. While a positive *KRAS* ctDNA result reliably reflects tumor-derived genomic alterations and provides strong rule-in value, a negative result cannot exclude the presence of malignancy. The variability across studies highlights the impact of biological factors, such as tumor burden and ctDNA shedding, as well as technical differences in analytical platforms and pre-analytical processing. Sensitivity demonstrated moderate heterogeneity (I^2^ = 59.0%), whereas specificity showed minimal heterogeneity (I^2^ = 4.5%). These findings highlight the need for prospective validation and methodological standardization.

Despite these limitations, *KRAS* testing using ctDNA represents a valuable complementary diagnostic tool that can support clinical decision-making, particularly when tissue sampling is challenging or insufficient. As sequencing technologies advance, error suppression improves, and integrated multi-omic approaches become more widely available, the sensitivity of ctDNA testing is expected to increase. Standardized methodologies are required for future prospective studies to refine its clinical implementation and evaluate its role within multimodal diagnostic pathways for lung cancer.

## Figures and Tables

**Table 1:** Summary of study characteristics. The table summarizes the key characteristics of the nine study arms included in the quantitative synthesis. It summarizes diagnostic contingency data—true positives (TPs), false positives (FPs), false negatives (FNs) and true negatives (TNs)—as well as the total number of participants (Total N) in each study arm.

Study	Mutation Panel	Platform	TPs	FPs	FNs	TNs	Total Number
Dawar et al. [13]	*KRAS*	ddPCR	47	13	18	42	120
Visser et al. [14]	*KRAS*	digital PCR	53	2	16	47	118
Bauml et al. [15]	*KRAS*	qPCR	82	0	34	73	189
Crucitta et al. [16]	*KRAS*	NGS	12	2	3	5	22
Xie et al. [17]	*KRAS*	PCR	5	0	1	65	71
Arhant et al. [18]	*KRAS*	ddPCR	21	0	13	0	34
Parisi et al. [19]	*KRAS*	digital PCR	10	0	2	15	27
Pathak et al. [20]	*KRAS*	qPCR	17	0	46	24	87
Qvick et al. [21]	*KRAS*	ddPCR	8	2	3	10	23

**Table 2:** Diagnostic accuracy metrics across individual study datasets. This table summarizes the sensitivity, specificity and diagnostic odds ratio (DOR) reported in studies that assessed the detection of *KRAS* mutations in ctDNA. While sensitivity varied substantially across datasets, specificity remained consistently high. An infinite DOR (∞) indicates that no false-positive results were observed in that study arm (i.e., specificity = 100%), which causes the odds ratio to be mathematically undefined and implies that the diagnostic performance for positive test results is extremely high.

Study	Sensitivity (%)	Specificity (%)	DOR
Dawar et al. [13]	72.3	75.9	8.08
Visser et al. [14]	76.4	95.9	61.6
Bauml et al. [15]	70.5	99.3	*∞*
Crucitta et al. [16]	78.1	68.8	7.857
Xie et al. [17]	78.6	99.2	*∞*
Arhant et al. [18]	61.8	-	*-*
Parisi et al. [19]	80.8	96.9	*∞*
Pathak et al. [20]	27.3	98.0	*∞*
Qvick et al. [21]	70.8	80.8	10.2

## Data Availability

The original contributions presented in this study are included in the article/Supplementary Material. Further inquiries can be directed to the corresponding author.

## Supplementary Materials

The following supporting information can be downloaded at: https://www.mdpi.com/article/10.3390/cancers18020250/s1, Figure S1: A summary of the risk of bias and applicability concerns based on the QUADAS-2 tool was created for the nine included diagnostic study arms [13,14,15,16,17,18,19,20,21]. Each study was evaluated across four domains: patient selection (D1); the index test (D2); the reference standard (D3); and flow and timing (D4). Overall risk of bias was also considered.

## Abbreviations

The following abbreviations are used in this manuscript:

ctDNA	Circulating tumor DNA
CTC	Circulating Tumor Cell
TKIs	Tyrosine kinase inhibitors
NSCLC	Non-small-cell lung cancer
PRISMA	Preferred Reporting Items for Systematic Reviews and Meta-Analyses
TP	True Positive
FP	False Positive
FN	False Negative
TN	True Negative
qPCR	Quantitative PCR
ddPCR	Droplet digital PCR
NGS	Next-generation sequencing
DORs	Diagnostic odds ratios
PPV	Positive predictive value
NPV	Negative predictive value
SROC	Summary receiver operating characteristic
CHIP	Clonal hematopoiesis of indeterminate potential
