# Supplementary material for: KRAS Mutations in Circulating Tumor DNA for Lung Cancer Diagnosis: A Comprehensive Meta-Analysis
Source: Cancers (Basel). 2026 Jan 14;18(2):250. doi: 10.3390/cancers18020250 (PMC12838994; doi:10.3390/cancers18020250)
Supplement: Supplementary file 1 [file cancers-18-00250-s001.zip › cancers-4076628-supplementary.pdf]

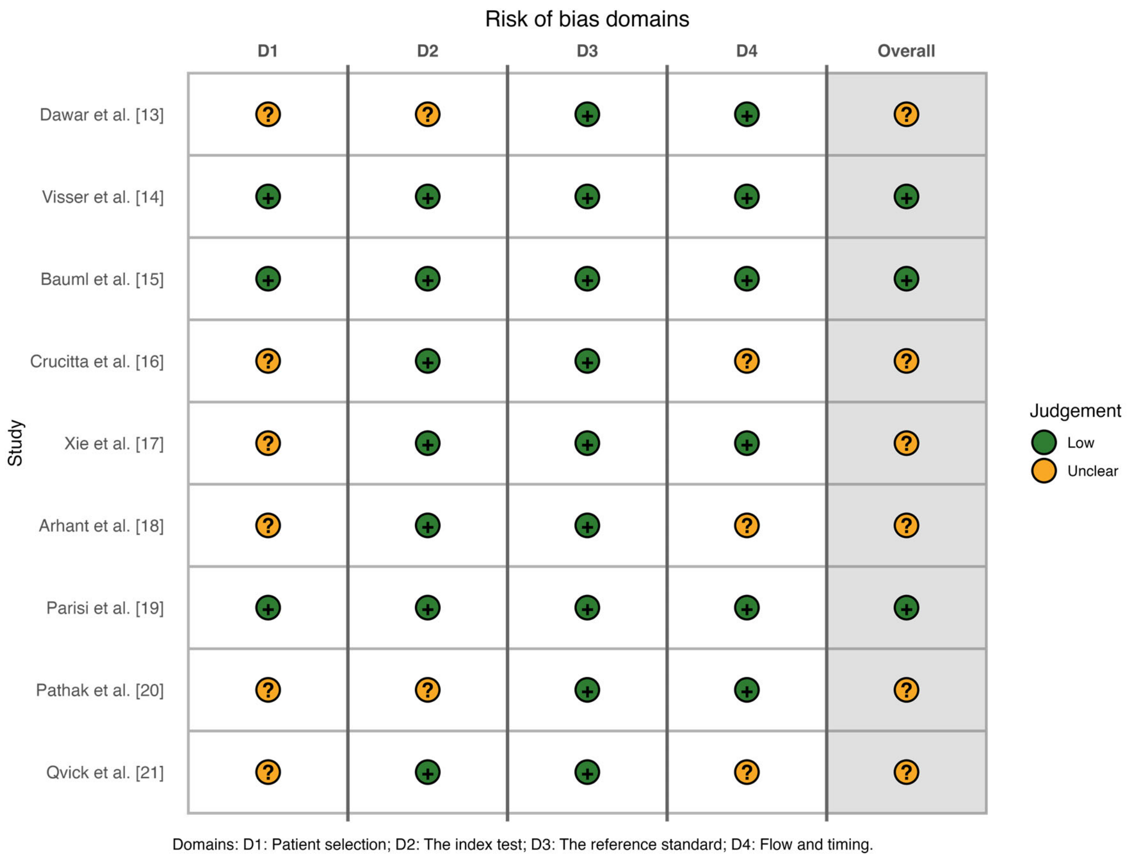

Figure S1. A summary of the risk of bias and applicability concerns based on the QUADAS-2 tool was created for the nine included diagnostic study arms [13–21]. Each study was evaluated across four domains: patient selection (D1); the index test (D2); the reference standard (D3); and flow and timing (D4). Overall risk of bias was also considered.
